# Speed limit of quantum metrology

**DOI:** 10.1038/s41598-023-39082-w

**Published:** 2023-07-25

**Authors:** Yusef Maleki, Bahram Ahansaz, Alireza Maleki

**Affiliations:** 1 Department of Physics and Astronomy, Texas A&M University, College Station, Texas, USA; 2 Department of Physics, Azarbaijan Shahid Madani University, Tabriz, Iran; 3 Department of Physics, Sharif University of Technology, Tehran, Iran

**Keywords:** Physics, Quantum physics, Quantum information, Quantum metrology

## Abstract

Quantum metrology employs nonclassical systems to improve the sensitivity of measurements. The ultimate limit of this sensitivity is dictated by the quantum Cramér–Rao bound. On the other hand, the quantum speed limit bounds the speed of dynamics of any quantum process. We show that the speed limit of quantum dynamics sets a fundamental bound on the minimum attainable phase estimation error through the quantum Cramér–Rao bound, relating the precision directly to the underlying dynamics of the system. In particular, various metrologically important states are considered, and their dynamical speeds are analyzed. We find that the bound could, in fact, be related to the nonclassicality of quantum states through the Mandel Q parameter.

Estimation of an unknown parameter is a central task in science and incorporates a broad class of applications, from gravitational wave detection^1,2^ to nanoscale superresolving microscopy^3^ and ultrasensitive spectroscopy^4^. The essential procedure in the estimation of an unknown parameter includes an inference from a set of data about the parameter to which they are attributed^5–7^. In parameter estimation procedures, the accuracy of estimation can be improved by repeating the experiment and collecting more information on the parameter. More precisely, using *N* independent resources, for measuring the parameter $\varphi$, the optimal sensitivity of the parameter is determined by the central limit theorem scaling as $\Delta \varphi \propto 1/{\sqrt{N}}$^8–10^. Quantum probes can beat the classical limit, enabling unprecedentedly enhanced accuracy with an extra $1/{\sqrt{N}}$ improvement of precision, when using *N* resources^9,11–14^. Interestingly, such improvements on the estimation and sensing are fundamentally restricted by the underlying physics of the probe systems, which dictates a bound on the ultimate attainable precision of the estimated parameter. This fundamental bound in the estimation process is usually given via the Cramér–Rao bound (CRB)^15^. For estimation of an unknown parameter $\varphi$, the unbiased quantum CRB is defined as^4^

1 $$\begin{aligned} \delta \varphi _{CRB}= 1/{\sqrt{\mathscr {F}_Q(\varphi )}}, \end{aligned}$$

where $\mathscr {F}_Q(\varphi )=Tr[\rho (\varphi ) L_{\varphi }^2]$ is the quantum Fisher information^4,10^. The symmetric logarithmic derivative $L_{\varphi }$ is defined as $\partial _{\varphi } \rho (\varphi )=(1/2)[\rho (\varphi ) L_{\varphi }+L_{\varphi }\rho (\varphi ) ]$, with $\rho (\varphi )$ being the density matrix. If the pure state $|\Psi \rangle$ is used as the quantum probe, the Fisher information of the quantum state $|\Psi _\varphi \rangle$ gives^7,10^

2 $$\begin{aligned} \mathscr {F}_Q(\varphi )=4(\langle \partial _\varphi \Psi _\varphi |\partial _\varphi \Psi _\varphi \rangle -|\langle \Psi _\varphi |\partial _\varphi \Psi _\varphi \rangle |^2). \end{aligned}$$

On the other hand, determining the speed of the quantum dynamics of a system is a pivotal task in many physical domains^16,17^. The faster dynamics in quantum gates can expedite computation^18–20^. Also, in quantum control, more rapid evolution assists in suppressing decoherence by shortening the evolution time^20–22^. In condensed matter physics, for determining how fast correlations can be spread in quantum many-body systems, understanding the dynamical speed is required^23–25^. Quantum speed limit (QSL) dictates a fundamental bound on the speed of evolution of all such quantum processes^26–34^. For a closed system, Mandelstam and Tamm derived the first expression ${\pi \hbar }/({2 \Delta H})$ for the QSL for the systems evolving between two orthogonal states, resulting in the modern interpretation of the time-energy uncertainty principle^35^. Later, Margolus and Levitin proposed the alternative expression ${\pi \hbar }/{2\langle H\rangle }$ for such quantum dynamics^36^. These two bounds, known as MT and ML bound respectively, determine the minimum time that a system needs to evolve from its initial state to its final orthogonal state through

3 $$\begin{aligned} \tau _{QSL}={\textrm{max}}\left( { \frac{\pi }{2 \Delta H}, \frac{\pi }{2\langle H\rangle }}\right) . \end{aligned}$$

where $\Delta H$ is the variance of the Hamiltonian of the system, and $\langle H\rangle$ is the expectation value of the Hamiltonian with respect to the initial state. For the evolution between two nonorthogonal states, a generalized form of the above formula was defined such that^37^

4 $$\begin{aligned} \tau _{QSL}=\textrm{max}\{\mathscr {T}_{MT},\mathscr {T}_{ML}\}, \end{aligned}$$

where $\mathscr {T}_{MT}= \frac{\hbar }{\Delta H} \mathscr {L}(\rho _0,\rho _T)$ and $\mathscr {T}_{ML}=\frac{2\hbar }{\pi \langle H \rangle } \mathscr {L}^{2}(\rho _0,\rho _T)$ are the generalized Mandelstam–Tamm and Margolus–Levitin bounds, respectively. $\rho _0$ and $\rho _T$ are the density matrices of the initial (at time equal to 0) and the final state of the system (at time equal to *T*), respectively. Moreover, $\mathscr {L}(\rho _0,\rho _T)$ is the Bures angle which determines the generalized angle between two arbitrary density matrices $\mathscr {L}(\rho _0,\rho _T)=\textrm{arccos}(\sqrt{F(\rho _0,\rho _T)})$^38^, with $F(\rho _0,\rho _T)$ being the fidelity between the two density matrices $\rho _0$ and $\rho _T$^39,40^

$$\begin{aligned} F(\rho _0,\rho _T)=\Big [ tr \Big \{\sqrt{\sqrt{\rho _0} \rho _T \sqrt{\rho _0} } \Big \} \Big ] ^2. \end{aligned}$$

As we know quantum metrology involves building measuring devices which develop incredibly precise measuring devices. In addition, increasing the quantum speed limit of one such device would allow for faster measurements which could theoretically yield more accurate results. For this reason, it is an essential task to highlight the interplay of the quantum metrology and the speed of quantum evolution. For determining the underlying dynamical structure of quantum systems and their usefulness for quantum metrology, the relationship between the quantum Fisher information (and therefore metrology) and quantum speed limits was clearly elucidated in Ref.^41^. Moreover, the relation between quantum metrology and different quantum speed limits has been further explored in several works^42–47^. In this paper, by considering various metrologically important states, we show how the speed limit of quantum dynamics, given by the generalized Mandelstam–Tamm and Margolus–Levitin bounds, provides a fundamental bound on the attainable phase estimation error bound dictated by the CRB through a quantum probe in interferometry. The result of this research could lead to devices with faster detection rates and improved accuracy.

## Speed limit of quantum metrology

To understand the relation between phase estimation error bound and speed of the quantum dynamics of a system we start with the coherent state $\vert \alpha \rangle =e^{-{\mid \alpha \mid }/{2}} \sum _{n=0}^\infty \frac{\alpha ^n}{\sqrt{n!}}\vert n \rangle$^48^, for illustration. The Hamiltonian of the system can be expressed as $H=\hbar \omega a^{\dagger }a$, where *a* and $a^{\dagger }$ are the annihilation and creation operators acting on the Fock basis of the photons. For the system undergoing time evolution with respect to the Hamiltonian *H*, the unitary operator is given by $U(t)=e^{-i\omega \Delta t a^{\dagger }a}$, where $\Delta t=t-t_0$ is the time interval of the unitary evolution of the system. Thus, by defining the phase shift $\varphi$ such that $\omega \Delta t = \varphi$, the time evolution operator degenerates to $U(\varphi )=e^{-i\varphi a^{\dagger }a}$, which is identical to the unitary operator of the phase shift in the interferometry. Based on this Hamiltonian, the coherent state $\vert \alpha \rangle$ evolves to another coherent state given by $\vert e^{-i\varphi } \alpha \rangle$; which is not orthogonal to its initial state $\vert \alpha \rangle$ in general, for any nonzero $\varphi$. Therefore, by defining $\Delta \varphi _{MT}=\omega \mathscr {T}_{MT}$ and $\Delta \varphi _{ML}=\omega \mathscr {T}_{ML}$ the bounds in Eq. (4) are found to be

5 $$\begin{aligned} \Delta \varphi _{MT}&=\frac{1}{\mid \alpha \mid } \textrm{arccos}(e^{{-|\alpha |^2}(1-\cos {\varphi })}), \nonumber \\ \Delta \varphi _{ML}&=\frac{2}{\pi \mid \alpha \mid ^2} \textrm{arccos}^2(e^{{-|\alpha |^2}(1-\cos {\varphi })}). \end{aligned}$$

On the other hand, using quantum Fisher information formula in Eq. (2), the Cramér–Rao bound of the coherent state reads $\delta \varphi _{CRB}=1/(2|\alpha |)$. Hence, considering Eq. (5), we arrive at

6 $$\begin{aligned} \delta \varphi _{CRB}&\geqslant \frac{\Delta \varphi _{MT}}{2 \textrm{arccos}(e^{{-2|\alpha |^2}}) },\nonumber \\ \delta \varphi _{CRB}&\geqslant \sqrt{\frac{\pi }{8}\frac{\Delta \varphi _{ML}}{\textrm{arccos}^2(e^{{-2|\alpha |^2}}) } }. \end{aligned}$$

Thus, the phase estimation error with the coherent state is bounded though $\Delta \varphi _{MT}$ and $\Delta \varphi _{ML}$. From Eq. (6) we immediately find

7 $$\begin{aligned} \delta \varphi _{CRB}> \frac{\Delta \varphi _{MT}}{\pi },\nonumber \\ \delta \varphi _{CRB} > \sqrt{\frac{\Delta \varphi _{ML}}{2\pi } }. \end{aligned}$$

Therefore, for a coherent state, the ultimate achievable error given by the CRB, is fundamentally bounded by the speed of the dynamical evolution of the quantum state.

Similarly, using Eqs. (4) and (5) the upper bound of the $\Delta \varphi _{QSL}$ is given by

8 $$\begin{aligned} \Delta \varphi _{QSL}&\le \textrm{max}\{\frac{\pi }{2\mid \alpha \mid },\frac{\pi }{2\mid \alpha \mid ^2}\} =\textrm{max}\{\pi \delta \varphi _{CRB},2\pi \delta \varphi _{CRB}^2 \}. \end{aligned}$$

Therefore, the lower bound on the CRB of the coherent state in terms of the QSL phase $\Delta \varphi _{QSL}$ is given by

9 $$\begin{aligned} {\left\{ \begin{array}{ll} \delta \varphi _{CRB}\ge \frac{1}{\pi }\Delta \varphi _{QSL} &{} \text {if } \!\begin{aligned} |\alpha |&{}\ge 1, \end{aligned} \\ \delta \varphi _{CRB}\ge \sqrt{\frac{1}{2\pi }\Delta \varphi _{QSL}} &{} \text {otherwise}. \end{array}\right. } \end{aligned}$$

This relation directly shows that the QSL dictates a lower bound on the ultimate limit of precision attainable by a coherent state in the interferometric phase estimation.

In the example above, we have investigated a single-mode coherent state in interferometry. However, it is interesting to consider entangled states due to their significant role in quantum-enhanced phase estimation, and metrology in general^9,11,12^. Thus, we investigate an entangled state which has been proven to be of substantial importance in quantum metrology given by^49^

10 $$\begin{aligned} |\Psi \rangle = \mathscr {N} (\vert \alpha \rangle \vert 0 \rangle +\vert 0 \rangle \vert \alpha \rangle ). \end{aligned}$$

Here, the normalization factor $\mathscr {N}$ is $\mathscr {N} =\frac{1}{\sqrt{2(1+e^{-|\alpha |^2})}}$. Thus, the Hamiltonian of the mode *i* can be expressed as $H_i=\hbar \omega a^{\dagger }_i a_i$ for $i=1,2$. We consider the system undergoing the time evolution with respect to the Hamiltonian of the second subsystem $H_2$, described by the unitary operator $U(t)=e^{-i\omega \Delta t a^{\dagger }_2a_2}.$ This operator translates into the unitary phase shift operator $U(\varphi )=e^{-i\varphi a^{\dagger }_2a_2}$. Thus, applying $U(\varphi )$ to the state $|\Psi \rangle$ gives

11 $$\begin{aligned} |\Psi _\varphi \rangle = \mathscr {N} (\vert \alpha \rangle \vert 0 \rangle +\vert 0 \rangle \vert e^{i\varphi }\alpha \rangle ). \end{aligned}$$

For the state $|\Psi \rangle$, the average photon number of the second mode is $\langle N_2 \rangle =\langle a^{\dagger }_2a_2 \rangle =\mathscr {N}^2 |\alpha |^2$. Hence, the variance of the photons of the second mode is found as $\langle \Delta N_2 \rangle =\mathscr {N}^2( |\alpha |^2+ |\alpha |^4)-\mathscr {N}^4 |\alpha |^4.$ Furthermore, the fidelity between the time evolved and the initial coherent states is

12 $$\begin{aligned} F(\rho _0,\rho _T)&=\mathscr {N}^4\Big [(1+2 e^{-|\alpha |^2})^2+ e^{-2|\alpha |^2 (1-\cos \varphi )}+2(1+2 e^{-|\alpha |^2}) e^{-|\alpha |^2 (1-\cos \varphi )} \cos (|\alpha |^2 \sin \varphi )\Big ]. \end{aligned}$$

The minimum of the fidelity depends on both $|\alpha |$ and $\varphi$. Thus, unlike the previous example, there is no single $\varphi$ minimizing fidelity for all the given parameter $|\alpha |$. The minimum value of the fidelity can be calculated numerically for specific values of $|\alpha |$. Nevertheless, we always have $F(\rho _0,\rho _T)>\mathscr {N}^4\big [ 1+2 e^{-|\alpha |^2}-e^{-2|\alpha |^2} \big ]^2.$ Hence, the Bures angle is bounded from above via $\ell (\alpha )=\textrm{arccos}\Big [\frac{1+2 e^{-|\alpha |^2}-e^{-2|\alpha |^2}}{2(1+e^{-|\alpha |^2})}\Big ]$. Thus, we arrive at $\mathscr {L}(\rho _0,\rho _T)<\ell (\alpha )< \textrm{arccos}(1/2)=\pi /3.$ On the other hand, using the quantum Fisher information formula, the CRB is

13 $$\begin{aligned} \delta \varphi _{CRB} =\dfrac{1}{2\mathscr {N} |\alpha | \sqrt{\big [ 1+ |\alpha |^2 (1-\mathscr {N}^2 ) \big ]}}. \end{aligned}$$

Therefore, in terms of $\Delta \varphi _{MT}$, CRB is limited by

14 $$\begin{aligned} \delta \varphi _{CRB}&\geqslant \frac{\Delta \varphi _{MT}}{2 \textrm{max}( \mathscr {L}(\rho _0,\rho _T)) }>\frac{\Delta \varphi _{MT}}{2 \ell (\alpha )}. \end{aligned}$$

Thus, we can express the MT bound of the phase estimation as $\delta \varphi _{CRB}>(3/2 \pi ){\Delta \varphi _{MT}}$. If we further loosen the bound, we can arrive at $\delta \varphi _{CRB}>{\Delta \varphi _{MT}}/\pi$, akin to the bound of a single coherent state.

Now, considering $\Delta \varphi _{ML}$ we arrive at

15 $$\begin{aligned} \delta \varphi _{CRB}&\geqslant \frac{1}{\sqrt{1+ |\alpha |^2 (1-\mathscr {N}^2 ) }}\sqrt{\frac{\pi }{8}\frac{\Delta \varphi _{ML}}{ \textrm{max}( \mathscr {L}(\rho _0,\rho _T))^2 }} \mathbf{}\nonumber \\&>\frac{1}{\sqrt{1+ |\alpha |^2 (1-\mathscr {N}^2 ) }}\sqrt{\frac{\pi }{8}\frac{\Delta \varphi _{ML}}{ \ell (\alpha )^2 }} \nonumber \\&>\frac{1}{\sqrt{1+ |\alpha |^2 (1-\mathscr {N}^2 ) }}\sqrt{\frac{9}{8}\frac{\Delta \varphi _{ML}}{ \pi }}. \end{aligned}$$

Therefore, the CRB is limited by

16 $$\begin{aligned} \delta \varphi _{CRB} >\frac{1}{\sqrt{1+ |\alpha |^2 (1-\mathscr {N}^2 ) }}\sqrt{\frac{\Delta \varphi _{ML}}{2\pi }}. \end{aligned}$$

As mentioned before, the right hand side of the MT bound for a single coherent state is equal to ${\Delta \varphi _{MT}}/\pi$. However, it is different from the ML bound for a single coherent state where we found $\delta \varphi _{CRB}\ge \sqrt{\frac{\Delta \varphi _{ML}}{2\pi }}$. Thus, with these analyses and using Eq. (4), we introduce the ultimate phase estimation bound dictated by $\Delta \varphi _{MT}$, for state $\rho _0$ as

17 $$\begin{aligned} \delta \varphi _{CRB}&\geqslant \frac{\Delta \varphi _{MT}}{2 \mathscr {L}(\rho _0,\rho _T) }\geqslant \frac{1}{\pi }\Delta \varphi _{MT}. \end{aligned}$$

And, the ultimate phase estimation bound dictated by $\Delta \varphi _{ML}$ as

18 $$\begin{aligned} \delta \varphi _{CRB}&\geqslant \frac{1}{\sqrt{1+ Q_M }}\sqrt{\frac{\pi }{8}\frac{\Delta \varphi _{ML}}{ \mathscr {L}(\rho _0,\rho _T)^2 }} \geqslant \frac{1}{\sqrt{1+ Q_M }}\sqrt{\frac{\Delta \varphi _{ML}}{ 2\pi }}. \end{aligned}$$

Here, $Q_M$ is the so-called Mandel Q parameter expressed as $Q_M=\dfrac{\langle \Delta n^2 \rangle -\langle n\rangle }{\langle n\rangle }$^50^. We note that the right hand side of Eqs. (17) and (18) are equal. This coincides with the fact that even though there are two different bounds for QSL, the phase estimation is limited by a uniquely defined single Cramér–Rao bound. It is quite interesting to note that $\Delta \varphi _{ML}$, is related to the statistics of the quantum probes through the Mandel Q parameter. Accordingly, sub-Poissonian statistics satisfy $Q_M<0$, and states with such statistics are known to be nonclassical. For Poissonian statistics $Q_M=0$, which is relaxed by the coherent states. For states having Poissonian or sub-Poissonian statistics, the phase estimation bound given by Eq. (18) can be further loosened to get

19 $$\begin{aligned} \delta \varphi _{CRB} \geqslant \sqrt{\frac{\pi }{8}\frac{\Delta \varphi _{ML}}{ \mathscr {L}(\rho _0,\rho _T)^2 }}\geqslant \sqrt{\frac{\Delta \varphi _{ML}}{ 2\pi }}. \end{aligned}$$

This recovers the coherent state bound, given by Eq. (7). For the states with super-Poissonian statistics $Q_M>0$, the ML bound cannot be reduced to Eq. (19), in general. Hence, the ML bound of the entangled coherent state in Eqs. (15) and (16) can be understood by noting that the Mandel Q parameter of this state is $Q_M= |\alpha |^2 (1-\mathscr {N}^2 )$. Thus, when the average number of photons is large enough ($\bar{n}= |\alpha |^2>> 1$), the phase estimation bound is $\delta \varphi _{CRB} \gtrsim \sqrt{\frac{\Delta \varphi _{ML}}{\bar{n}\pi }}$. The bounds introduced in Eqs. (17) and (18) are generic and can be applied to a vast class of quantum states beyond the coherent states. To see this and to further exemplify the utility of the bounds obtained here, we apply the QSL bounds to the squeezed states metrology in the Methods section. Hence, from these analyses we realize that the states which minimize CRB, inevitably need to maximize QSL. This agrees with the observation of Ref.^42^, where a quantum metrological setting, in the context of a particular non-Markovian quantum evolution of two two-level atoms, is considered.

For a given *N* photon interferometry, the ultimate precision reduces to the Heisenberg limit $\delta \varphi _{CRB}=1/N$, which is known to be relaxed by the N00N state^13,14^

20 $$\begin{aligned} \vert \psi \rangle =\frac{1}{\sqrt{2}}(\vert N \rangle _1 \vert 0 \rangle _2+\vert 0 \rangle _1 \vert N \rangle _2). \end{aligned}$$

This suggests that *N* photons can be in the first mode and no photon in the second mode or vice versa. Provided that the N00N state undergoes the phase shift $\varphi$ described by $U(\varphi )=e^{-i\varphi a^{\dagger }_2a_2}$, it degenerates to

21 $$\begin{aligned} \vert \psi _ \varphi \rangle =\frac{1}{\sqrt{2}}(\vert N \rangle _1 \vert 0 \rangle _2+e^{-i \varphi N}\vert 0 \rangle _1 \vert N \rangle _2. \end{aligned}$$

Thus, we immediately obtain the well–known result $\delta \varphi _{CRB}=1/N$. On the other hand, the minimum time that takes the N00N state to evolve to its orthogonal state is given by Eq. (3). Since, for the N00N state $\Delta H_2=\langle H_2\rangle =N/2$, we have $\tau _{QSL}={\pi }/{\omega N}$. From $\Delta \varphi _{QSL}=\omega \tau _{QSL}$, we find that $\Delta \varphi _{QSL}= {\pi }/{N}$. This agrees with the fact that, when $\varphi N=(2k+1) \pi$, the state in Eq. (21) becomes orthogonal to the N00N state in Eq. (20). Thus

22 $$\begin{aligned} \delta \varphi _{CRB} = \frac{1}{\pi } \Delta \varphi _{QSL} \quad \Rightarrow \quad \delta t_{CRB}=\frac{1}{\pi }\tau _{QSL} \end{aligned}$$

where, $\delta t_{CRB}=({1}/{\omega })\delta \varphi _{CRB}$. Thus, N00N state is not only optimal for quantum metrology, but it is also optimal for QSL, evolving with the ultimate speed $\textit{v} \propto N/\pi$. Quantum estimation beyond the classical regime that can reach the HL of precision is not well explored in the experiments, and most of such studies are limited to photon number *N*. Considering the role of the QSL in dynamical features of the quantum systems, understanding the relation between QSL and CRB can play a central role in enhancing the phase estimation precision, e.g., by quantum control techniques.

It is worth mentioning that our studies for coherent state, entangled coherent state and NOON state, revealed that how the speed limit of quantum dynamics provides a fundamental bound on the attainable phase estimation error bound. A more interesting phenomenon here is that the accuracy of the estimation can be improved by increasing the speed of quantum evolution. These results highlight the fact that two seemingly unrelated concepts (CRB and QSL time) are deeply connected in a more fundamental trait.

## Numerical results

In our analyses so far, we considered pure quantum states and showed the relation between QSL and CRB through Eqs. (17) and (18). However, in a practical setting, generating and preserving pure quantum states are challenging from an experimental perspective. In most scenarios, quantum states become mixed as they inevitably interact with their surrounding environment^51^. In this section, we consider the relation between QSL and CRB in the mixed state realm and address the characteristics of the bounds in Eqs. (17) and (18) in the mixed state scenario.

### General *d*-dimensional Werner state

The mixed states that we consider here are the class of states called generalized Werner states. The Werner state is an important type of mixed state that plays a fundamental role in the foundations of quantum mechanics and quantum information theory. The most natural generalization of the $2\times 2$ Werner states to the higher dimensions can be written as^52^

23 $$\begin{aligned} \rho =p| \psi \rangle \langle \psi |+(1-p)\frac{I}{d^{2}}, \end{aligned}$$

where the pure state $\vert \psi \rangle$ is defined as

24 $$\begin{aligned} \vert \psi \rangle =\alpha \vert N \rangle _1 \vert 0 \rangle _2+\beta \vert 0 \rangle _1 \vert N \rangle _2, \end{aligned}$$

and the normalization condition implies that $|\alpha |^2+|\beta |^2=1$. Here the dimension of Hilbert space is $d=N+1$. Once the above Werner state undergoes the phase shift $\varphi$, via the unitary operator $U(\varphi )=e^{-i\varphi a^{\dagger }_2a_2}$, it transforms into

25 $$\begin{aligned} \rho _ \varphi =p| \psi _ \varphi \rangle \langle \psi _ \varphi |+(1-p)\frac{I}{d^{2}}, \end{aligned}$$

where we have

26 $$\begin{aligned} \vert \psi _ \varphi \rangle =\alpha \vert N \rangle _1 \vert 0 \rangle _2+\beta e^{-i \varphi N} \vert 0 \rangle _1 \vert N \rangle _2. \end{aligned}$$

Now, in order to investigate the given bounds in Eqs. (17) and (18) we introduce the following quantities

27 $$\begin{aligned} B_1=\delta \varphi _{CRB}-\frac{\Delta \varphi _{MT}}{2 \mathscr {L}(\rho _0,\rho _T) }\ge 0, \end{aligned}$$

and similarly

28 $$\begin{aligned} B_2=\delta \varphi _{CRB}-\frac{1}{\sqrt{1+ Q_M }}\sqrt{\frac{\pi }{8}\frac{\Delta \varphi _{ML}}{ ( \mathscr {L}(\rho _0,\rho _T))^2 }}\ge 0. \end{aligned}$$

**Figure 1:**
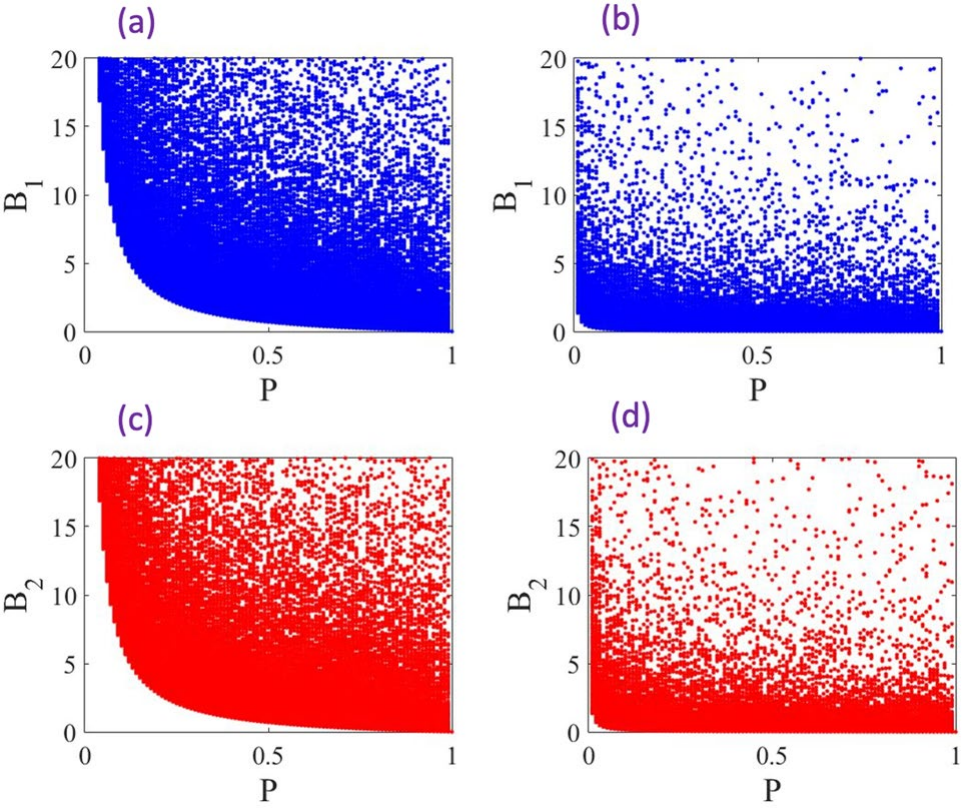
Numerical analyses of the inequalities (27) and (28) vs. *p*. Each plot presents $10^7$ random states, in total. Also, $\varphi =\pi /4$ is chosen to evaluate the inequalities. (**a**) and (**c**) present the inequalities for the photon number $N=1$, whereas (**b**) and (**d**) present the inequalities for the photon number $N=10$.

To analyze these bounds, we generate $10^5$ random states for each given fixed value of *p*, as presented in Fig. 1. The parameter *p* varies from 0 to 1 as determined by the state in Eq. (23), and each plot presents $10^7$ random states in total. The random states are generated through variations of $\alpha$ and $\beta$. It should also be noted that Fisher information for a given density matrix is independent of the phase shift $\varphi$; however, QSL directly depends on the phase shift. The inequalities (27) and (28) are valid for any given phase that inters the formulations of the QSL. In our analyses, we choose $\varphi =\pi /4$ for attaining the QSL terms. We present the performance of (27) in Fig. 1a and b for $N=1$ and $N=10$, respectively. A similar analysis for (28) is presented in Fig. 1c,d. Figure 1c presents the inequality for $N=1$ and Fig. 1d presents the inequality for $N=10$. As Fig. 1 clearly demonstrates, both inequalities (27) and (28) present similar features. As is readily seen from these plots, the bounds of $B_1$ and $B_2$ are tighter when $p=1$, where from Eq. (2) we find that the inequalities in the Eqs. (27) and (28) turn into equalities for the pure states. Hence, it should be emphasized that for states with high purities, $B_1$ and $B_2$ are small, while for states that are far from the set of pure states, the difference can be much larger. Also, the inequalities are tighter for the photon number $N=10$ in comparison to $N=1$. This suggests that increasing the number of photons *N* can also lead to tighter bounds in the considered setting.

### Single-qubit with various phase operations

In our analyses above, we considered pure and mixed quantum states. The phase generation was implemented by the unitary operator $U(\varphi )=e^{-i\varphi a^{\dagger }_2a_2}$ for both pure states and mixed states. In other words, the generator of the phase is $N_2=a^{\dagger }_2a_2$ in this framework. A natural question is how the bounds perform when the phase generator operator is something different than $N_2$. To address this, we consider the bounds for a single-qubit system, when the phase is implemented with various operators. The single-qubit system is described by a single-qubit spin operator $J_{\vec {n}}$ with a general unit vector $\vec {n}$. In fact, $J_{\vec {n}}$ is a pseudospin angular momentum operator given by

29 $$\begin{aligned} J_{\vec {n}}=\sum _{\alpha =x,y,z}\frac{1}{2} n_{\alpha }\sigma _{\alpha }=\frac{1}{2} \vec {n}\cdot \vec {\sigma }, \end{aligned}$$

where the vector $\vec {n}=(n_{x},n_{y},n_{z})$ is a unit vector and $\sigma _{\alpha }=(\alpha = x, y, z)$ are the Pauli matrices.

An arbitrary single-qubit state can be represented in the Bloch sphere as

30 $$\begin{aligned} \rho =\frac{1}{2} (I+\vec {r}\cdot \vec {\sigma }), \end{aligned}$$

where $\vec {r}=(r_{x},r_{y},r_{z})$ is the Bloch vector. Now, if the parametrization is described by the unitary operator $U(\varphi )=e^{-i\varphi J_{\vec {n}}}$, the output state can be given by $\rho (\varphi )=U(\varphi )\rho U^\dag (\varphi )$, where $\rho$ is an initial probe state.

**Figure 2:**
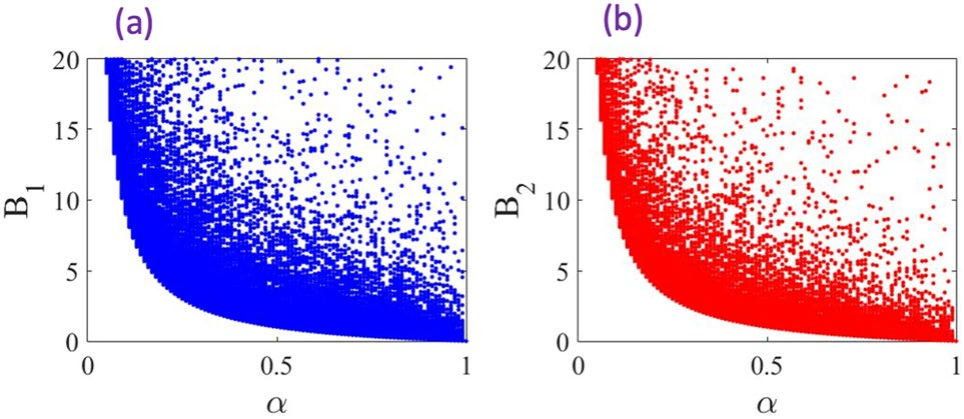
Numerical analyses of the inequalities (27) and (28) vs. $\alpha$. The plots presents $10^5$ random unit vector $\vec {n}=(n_{x},n_{y},n_{z})$ for each fixed value of $\alpha$. Thus, each plot presents $10^7$ random points in total. Also, $\varphi =\pi /4$ is chosen to evaluate the inequalities. The initial probe state is taken to be $\vec {r}=(\alpha ,0,0)$.

The result of the simulation for the random phase generating operator $J_{\vec {n}}$ is presented in Fig. 2. In these simulations we consider the Bloch vector of the initial probe state to be $\vec {r}=(\alpha ,0,0)$, where $\alpha =0$ corresponds to the maximally mixed state and $\alpha =1$ corresponds to the pure state $\rho =|\phi \rangle \langle \phi |$ with $|\phi \rangle =(|0 \rangle +|1 \rangle )/\sqrt{2}$. The simulation is performed for by assigning $10^5$ random values to the direction unit vector $\vec {n}=(n_{x},n_{y},n_{z})$ for each fixed $\alpha$. Therefore, each plot presents $10^7$ points in total. Similar to analyses of the Werner states the inequalities $B_{1}$ and $B_{2}$ become tighter by increasing $\alpha$. Whereas the bounds diverge for maximally mixed states, as expected.

## Conclusion

In conclusion, quantum Cramér–Rao bound imposes the ultimate limit of precision on metrology. On the other hand, the quantum speed limit dictates a fundamental upper bound on the speed of the dynamical evolution of any quantum process. Considering different important cases, we showed that the speed limit of quantum dynamics sets fundamental bounds on the attainable minimum error in the quantum phase estimation through Cramér–Rao bound. The quantum speed limit has revealed that the time-energy uncertainty principle, contrary to its old interpretation, is not a statement about simultaneous measurements. Rather, it is about the intrinsic time scale of the quantum evolution, interpreted as the time a quantum system needs to evolve from an initial to a final orthogonal state. Our results reveal a fundamental connection between the uncertainty in the measurement on the one hand and the intrinsic time scale of the unitary quantum evolution on the other. As an interesting conclusion, we demonstrated that increasing the speed of quantum evolution can improve the accuracy of the estimation. Beyond its fundamental relevance, this can be useful in quantum metrology, quantum control, and quantum information sciences.

## Methods

Here we consider the squeezed vacuum state as an example and find the connection between CRB and QSL time of the state. The squeezed vacuum state is defined as^50^

$$\begin{aligned} |\xi \rangle \ = \ S(\xi )|0\rangle , \end{aligned}$$

where $S(\xi )$ is the squeezing operator such that

$$\begin{aligned} S(\xi )\ =\ e^{-1/2 (\xi {a^{\dagger }}^2-\xi ^*a^2)}. \end{aligned}$$

The squeezing operator fulfills

$$\begin{aligned} S(\xi )S^{\dagger }(\xi ) \ = \ S^{\dagger }(\xi )S(\xi ) = \text {I}. \end{aligned}$$

Defining $\xi \ =\ r\ e^{i\varphi }$, we have the transformed operators of the system as^50^

$$\begin{aligned} S^{\dagger }(\xi )\ a \ S(\xi ) \ {}= & {} \ a \ \cosh r - a^{\dagger }\ e^{i\varphi } \sinh r, \\ S^{\dagger }(\xi )\ a^{\dagger } \ S(\xi ) \ {}= & {} \ a^{\dagger } \cosh r - a\ e^{-i\varphi } \sinh r. \end{aligned}$$

Therefore, the average number of photons in the squeezed vacuum can be given by

$$\begin{aligned} \langle a^{\dagger } a \rangle \ = \ \langle 0 |S^{\dagger }(\xi ) a^{\dagger } a S(\xi )| 0\rangle \ = \ \sinh ^2r. \end{aligned}$$

And similarly,

$$\begin{aligned} \langle ( a^{\dagger } a )^2\rangle \ = \ 3 \sinh ^4r + 2 \sinh ^2r. \end{aligned}$$

Equivalently, we have

$$\begin{aligned} \langle H \rangle \ = \ \hbar \omega \ \sinh ^2 r. \end{aligned}$$

And,

$$\begin{aligned} \Delta H \ = \ \sqrt{2}\ \hbar \omega \ \sinh r \cosh r. \end{aligned}$$

For an squeezed state, the unitary time evolution operator is $e^{-iHt/\hbar } \ = \ e^{-i \varphi a^{\dagger } a}$. Thus, time evolution of the initial squeezed state gives

$$\begin{aligned} e^{-iHt/\hbar } |\xi \rangle \ = \ e^{-i \varphi a^{\dagger } a} |\xi \rangle \ = \ |\xi e^{-2i \varphi }\rangle \end{aligned}$$

Thus, the fidelity can be calculated as

$$\begin{aligned} F = |\langle \xi |\xi e^{-2i\varphi }\rangle |^2=\dfrac{1}{\sqrt{\cosh ^4 r + \sinh ^4 r - 2 \cosh ^2 r \sinh ^2 r \cos (2\varphi )}}. \end{aligned}$$

Also, the quantum Fisher information $\mathscr {F}_Q$ reads

$$\begin{aligned} \mathscr {F}_Q \ = \ 4 \ \langle \xi |(\Delta H)^2|\xi \rangle \ = \ 8 \sinh r \cosh r. \end{aligned}$$

Thus, $(\Delta \varphi )_{CRB}$ can be obtained as

$$\begin{aligned} (\Delta \varphi )_{CRB} \ = \ \dfrac{1}{\sqrt{8}} \ \dfrac{1}{\sinh r \cosh r}. \end{aligned}$$

With these analyses, we have

$$\begin{aligned} \omega \tau _ {MT} = \dfrac{1}{\sqrt{2} \ \sinh r \cosh r} \ \mathscr {L}(\rho _0,\rho _\tau )=2 \ (\Delta \varphi )_{CRB} \ \mathscr {L}(\rho _0,\rho _\tau ). \end{aligned}$$

Thus, one has

$$\begin{aligned} (\Delta \varphi )_{CRB} \ \geqslant \ \dfrac{1}{\pi } (\Delta \varphi )_{MT}. \end{aligned}$$

On the other hand

$$\begin{aligned} (\Delta \varphi )_{ML} \ = \ \dfrac{2}{\pi } \ \dfrac{1}{\sinh ^2 r} \ \mathscr {L}^2(\rho _0,\rho _\tau ). \end{aligned}$$

However, from the equation of $(\Delta \varphi )_{CRB}$ we have

$$\begin{aligned} \dfrac{1}{\sinh ^2 r} \ = \ 8 \ \cosh ^2 r \ (\Delta \varphi )^2_{CRB}. \end{aligned}$$

Thus $$(\Delta \varphi )_{ML}$$ reads

$$\begin{aligned} (\Delta \varphi )_{ML} = \dfrac{2}{\pi } \ (8\cosh ^2 r \ (\Delta \varphi )^2_{CRB}) \ \mathscr {L}^2(\rho _0,\rho _\tau ) \leqslant \ \dfrac{\pi }{2}\ (8\cosh ^2 r \ (\Delta \varphi )^2_{CRB}). \end{aligned}$$

Therefore, we arrive at

$$\begin{aligned} (\Delta \varphi )_{CRB} \ \ge \ \dfrac{1}{\sqrt{2} \ \cosh r} \ \sqrt{\dfrac{(\Delta \varphi )_{ML}}{ 2\pi }}. \end{aligned}$$

On the other hand, for the squeezed vacuum, we have the Mandel *Q* parameter such that

$$\begin{aligned} 1+ Q_M \ = \ \dfrac{{\langle \Delta n \rangle } ^2}{\langle n \rangle } \ = \ 2 \ \cosh ^2 r. \end{aligned}$$

Thus, the lower bound of $(\Delta \varphi )_{CRB}$ can be expressed in terms of $(\Delta \varphi )_{ML}$ as

$$\begin{aligned} (\Delta \varphi )_{CRB} \ \ge \ \dfrac{1}{\sqrt{1+ Q_M }} \ \sqrt{\dfrac{(\Delta \varphi )_{ML}}{ 2\pi }}. \end{aligned}$$

## Data Availability

The data for the simulation results of the present study are available from the corresponding author upon a reasonable request.
